# End-Stage Heart Failure in Cardiac Sarcoidosis

**DOI:** 10.1161/CIRCULATIONAHA.123.066962

**Published:** 2024-03-12

**Authors:** Diana Velikanova, Pauli Pöyhönen, Jukka Lehtonen, Piia Simonen, Valtteri Uusitalo, Tapani Vihinen, Kari Kaikkonen, Petri Haataja, Tuomas Kerola, Tuomas T. Rissanen, Ville Vepsäläinen, Aleksi Alatalo, Päivi Pietilä-Effati, Markku Kupari

**Affiliations:** Heart and Lung Center (D.V., P.P., J.L., P.S., M.K.), Helsinki University Hospital and University of Helsinki, Finland.; Radiology (P.P., V.U.), Helsinki University Hospital and University of Helsinki, Finland.; Physiology and Nuclear Medicine (V.U.), Helsinki University Hospital and University of Helsinki, Finland.; Heart Center, Turku University Hospital, Finland (T.V.).; Medical Research Center Oulu, University and University Hospital of Oulu, Finland (K.K.).; Heart Hospital, Tampere University Hospital, Finland (P.H.).; Department of Internal Medicine, Päijät-Häme Central Hospital, Lahti, Finland (T.K.).; Heart Center, North Karelia Central Hospital, Joensuu, Finland (T.T.R.).; Heart Centre, Kuopio University Hospital, Kuopio, Finland (V.V.).; South Ostrobothnia Central Hospital, Seinäjoki, Finland (A.A.).; Vaasa Central Hospital, Vaasa, Finland (P.P-E.).

**Keywords:** cardiomyopathy, heart failure, sarcoidosis

Cardiac sarcoidosis (CS) is a rare inflammatory cardiomyopathy notorious for high and potentially fatal arrhythmogenicity.^1^ Manifest heart failure (HF) is not uncommon, and extensive granulomas and myocardial scarring can eventuate in end-stage HF (ESHF), requiring transplantation for survival.^1^ The incidence and predictors of ESHF in CS are poorly described.

We analyzed 512 cases from our nationwide CS registry; all cases were diagnosed in Finland between 1988 and the end of 2019.^2,3^ All patients fulfilled current CS diagnostic criteria^4^ and 225 (44%) had diagnosis based on myocardial histology. As previously described,^2,3^ the registry includes data on patients’ demographics, cardiac manifestations, diagnostic imaging, laboratory studies, and details of treatment and adverse events. We focused here on the time from presentation of CS to ESHF, wherein presentation was the first medical contact for symptoms attributable to CS, and ESHF was specified as either referral for heart transplantation or death from HF. The causes of death were ascertained from clinical and autopsy reports. All events were recorded through the end of 2020. The CS registry study was approved by the national ethical review board in 2009 (STM/1219/2009). Written informed consent was obtained from patients alive at the time of recruitment. The data cannot be made available to other researchers for purposes of reproducing the results because of restrictions related to the patient consent. Individual-level data cannot be shared openly.

Cause-specific cumulative incidence analysis was used to construct time-to-ESHF curves and to calculate incidence estimates; the Gray test was used for group comparisons. Noncardiac death and sudden cardiac death were considered competing events. Factors predictive of ESHF were analyzed by Fine and Gray models^5^ yielding subdistribution hazard ratios (SHR) with 95% CI. Analyses were performed using the SPSS v.25.0 (IBM Corporation, USA) and R (v4.0.4; R Foundation, Vienna, Austria).

At CS presentation, the patients’ median age (interquartile range) was 52 years (43–58 years), and 70% (356 of 512) were women. The main presenting manifestations were atrioventricular block (n=267 patients [52%]), sustained ventricular tachyarrhythmia (n=90 [18%]), and congestive HF (n=82 [16%]). The remaining patients (73 [14%]) presented with premature beats or symptoms such as chest pain, syncope, and dyspnea. The median presenting left ventricular ejection fraction (LVEF) was 55% (45–60%). Elevated cardiac troponins (exceeding the contemporaneous reference range) were found in 52% of patients (243 of 471), and 55% (228 of 417) had elevated natriuretic peptides (brain natriuretic peptide >100 ng/L or NT-proBNP [N-terminal brain natriuretic pro-peptide] >400 ng/L). On positron emission tomography (n=133), 92% of patients had abnormal cardiac 18F-fluorodeoxyglucose uptake. Cardiac magnetic resonance imaging (n=220) revealed late gadolinium enhancement in 97% of patients; the medians of LVEF, right ventricular ejection fraction, and late gadolinium enhancement mass were 49% (40–58%), 54% (46–61%), and 14% (8–22%), respectively. Treatment-wise, 501 patients (98%) received immunosuppression, 391 patients (76%) underwent implantation of a cardioverter-defibrillator, and 130 (25%) received a cardiac resynchronization therapy device.

During follow-up (median, 6.1 years [3.7–9.5 years]), 6 patients died of terminal HF and 26 died from a competing death, while 31 were referred for transplantation. Of those referred for transplantation, 21 underwent transplant surgery and 2 died on the waiting list. Of the 8 nonlisted patients, 4 had contraindications (2 received a left ventricular assist device for destination therapy), and 4 either died during the evaluation process (n=2) or awaited its completion at the closure of our study. The Figure [A] shows the time-to-ESHF graph and calculated incidence estimates. Univariable predictors of ESHF included manifest HF at presentation with a SHR of 2.86 (1.41–5.78) versus all other presenting manifestations (*P*=0.004), LVEF with a SHR of 0.58 per every 10% (0.45–0.76; *P*<0.001), elevated cardiac troponins with a SHR of 3.59 (1.61–8.00; *P*=0.002), and elevated natriuretic peptides with a SHR of 4.64 (1.37–15.70; *P*=0.014). The Figure [B–D] compares incidence graphs among patients and is stratified by presenting LVEF, cardiac troponins, and natriuretic peptides. Of the cardiac magnetic resonance imaging variables, late gadolinium enhancement mass (n=198) predicted ESHF with an SHR of 1.44 per every 5% (1.01–2.05; *P*=0.044) and right ventricular ejection fraction (n=209) with an SHR of 0.47 per every 10% (0.24–0.93; *P*=0.029). In a multivariable Fine and Gray model involving 471 patients with 31 ESHF events and 22 competing deaths, the predictors were LVEF with an SHR of 0.66 per every 10% (0.48–0.90; *P*=0.009) and elevated troponins with a SHR 2.89 (1.24–6.74; *P*=0.014), whereas presentation with HF was not independently predictive (*P*=0.350). LVEF and cardiac troponins had an additive predictive capacity: the 15-year incidence of ESHF was 25% (9–41%) in patients having both elevated troponins and LVEF ≤50% (n=130; 18 events) but only 2% (0–7%) in those with ejection fractions >50% and nonelevated troponins (n=162; 3 events).

**Figure.**
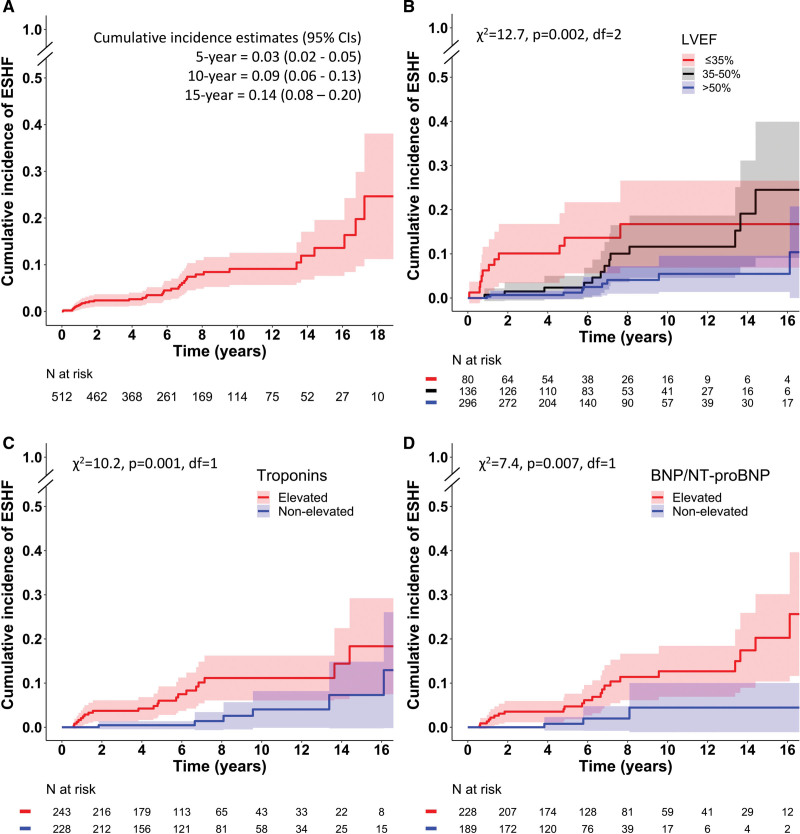
**Incidence graphs for end-stage heart failure in a 30-year Finnish cohort of biopsy-proven, clinically manifest cardiac sarcoidosis. A**, Cumulative incidence in the entire group of 512 patients. **B** through **D**, Comparisons of respective graphs across the subgroups of CS stratified by presenting echocardiographic left ventricular ejection fraction (**B**), elevation of cardiac troponins greater than the reference range (**C**), and elevation of either brain natriuretic peptide >100 ng/L or N-terminal natriuretic propeptide (NT-proBNP (N-terminal pro-B-type natriuretic peptide) >400 ng/L (**D**). *End-stage heart failure* was defined as referral for heart transplantation or death from terminal heart failure. Group comparisons were made using the Gray test. Shaded areas represent 95% CI.

We found that 14% of patients with clinically manifest CS lapsed into terminal HF within 15 years from presentation despite contemporaneous standard care. The risk of ESHF was associated with manifest HF at presentation and with laboratory and imaging biomarkers reflecting the extent of myocardial involvement. Importantly, having both LVEF ≤50% and elevated cardiac troponins at presentation predicted a 1 in 4 estimated 15-year risk of ESHF, however in their absence, the risk was much lower, at only 1 in 50.

## ARTICLE INFORMATION

### Acknowledgments

We thank our colleagues and staff in all participating hospitals for help with this study.

### Sources of Funding

This work was supported by a Finnish government grant for medical research, Aarne Koskelo’s foundation, and the Finnish Foundation for Cardiovascular Research. Dr Pöyhönen was supported by the Finnish Cultural Foundation and Finnish government grant for medical research.

### Disclosures

Dr Uusitalo reports advisory board activity with and lecture honoraria from GE Healthcare and Pfizer. Dr Lehtonen reports lecture honoraria from Pfizer. The other authors report no conflicts.
